# Sulforaphane‐Producing *Lacticaseibacillus paracasei* CCFM1221 Combining Broccoli Seed Extract Improved the Hepatoprotective Effect on Lipopolysaccharide‐Treated Mice

**DOI:** 10.1002/fsn3.72271

**Published:** 2026-08-24

**Authors:** Xin Tang, Yi Wei, Weiling Guo, Qiuxiang Zhang, Jianxin Zhao, Wei Chen, Bingyong Mao, Shumao Cui

**Affiliations:** ^1^ State Key Laboratory of Food Science and Resources Jiangnan University Wuxi China; ^2^ School of Food Science and Technology Jiangnan University Wuxi China; ^3^ National Engineering Research Center for Functional Food Jiangnan University Wuxi China; ^4^ International Joint‐Research Laboratory for Maternal‐Infant Microbiota and Health Jiangnan University Wuxi China

**Keywords:** broccoli seed extract, gut microbiota, hepatoprotective effect, *Lacticaseibacillus paracasei*, sulforaphane

## Abstract

This study aimed to investigate the effect of sulforaphane‐producing *Lacticaseibacillus paracasei* CCFM1221 on the hepatoprotective efficacy of broccoli seed extract (BSE) in lipopolysaccharide (LPS)‐treated mice. The results indicate that *L. paracasei* CCFM1221 exhibits a high metabolic capacity for glucoraphanin. The co‐administration of *L. paracasei* CCFM1221 and BSE could ameliorate liver index, serum biochemical indicators, and hepatic inflammatory cytokines by inhibiting the TLR4/NF‐κB pathway and activating the Nrf2 pathway. Compared with BSE alone, the combined intervention significantly suppressed the elevation of alanine transaminase (ALT), alkaline phosphatase (ALP), interleukin (IL‐1β), and MDA levels. Furthermore, the combination of *L. paracasei* CCFM1221 and BSE enhanced colonic sulforaphane levels from 195.63 ± 29.60 to 265.70 ± 34.29 nmol/g compared with BSE intervention alone. In addition, it elevated the abundance of short‐chain fatty acids (SCFAs)‐producing bacteria and promoted the growth of sulforaphane‐producing bacteria. These findings suggest that probiotics can enhance the activity of natural products, augmenting their pharmacological effects.

## Graphical abstract

*L. paracasei* CCFM1221 was capable of converting glucoraphanin into sulforaphane. In LPS‐induced mice, co‐administration of *L. paracasei* CCFM1221 with BSE (rich in glucoraphanin) alleviated liver injury. This combined intervention increased colonic sulforaphane levels and enriched gut bacteria involved in SCFAs and sulforaphane production. These changes were accompanied by suppression of hepatic TLR4/NF‐κB signaling and activation of the Nrf2 pathway, collectively contributing to the attenuation of liver injury. This study indicates that probiotics can potentiate the hepatoprotective efficacy of natural products.

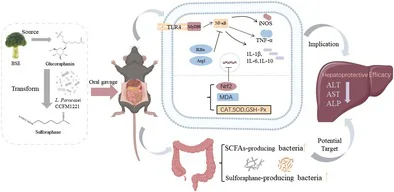

## 1 Introduction

Broccoli ( *Brassica oleracea* ), originating in the eastern Mediterranean region, has been widely introduced and cultivated in the United States, the United Kingdom, and China (Li, Mei, et al. 2019). In recent years, it has garnered significant attention due to its rich nutritional profile and diverse array of bioactive compounds, including glucoraphanin, polysaccharides, flavonoids, carotenoids, ascorbic acid, lutein, and various minerals. Epidemiological and functional studies have established a strong correlation between broccoli consumption and a reduced risk of chronic diseases such as diabetes, obesity, alcoholic liver disease, cancer, and vascular disorders (Li et al. 2022). Among these bioactive constituents, glucoraphanin, the primary glucosinolate found in broccoli seeds (West et al. 2004), serves as a precursor for active substances such as sulforaphane (1‐Isothiocyanato‐4‐(methylsulfinyl) butane). These metabolites exhibit potent anti‐tumor, anti‐inflammatory, antioxidant, hypoglycemic, and lipid‐lowering properties (Marshall et al. 2023). For instance, sulforaphane administration effectively reduces adipose tissue weight and prevents hepatic lipid droplet accumulation in high‐fat diet‐fed mice (Choi et al. 2014). It also prevents liver fibrosis development by activating the Nrf2 signaling pathway and suppressing the LPS/TLR4 signaling pathway (Ishida et al. 2021). However, significant interindividual differences exist in glucoraphanin's biotransformation to sulforaphane, resulting in variable therapeutic effects (Fahey et al. 2012). This biotransformation is strongly associated with gut microbiota composition (Liou et al. 2020). For example, *Bifidobacterium longum* CCFM1206 administration increased intestinal sulforaphane concentration, improving intestinal barrier function in colitis mice (Wu, Cui, et al. 2023). Additionally, *Lactiplantibacillus plantarum* KW30 and *Lactococcus lactis* subsp. lactis KF147 promote glucoraphanin conversion in broccoli extract, enhancing host exposure to bioactives (Mullaney et al. 2013).

Acute liver injury (ALI) is characterized by rapid hepatocyte function loss without prior symptoms. The mortality rate of ALI exceeds 50% (Guo et al. 2021). Excessive drug intake and ischemia/reperfusion have become major ALI morbidity factors. Drug overconsumption disrupts gut microbiota composition, potentially increasing intestinal (lipopolysaccharide) LPS concentration (Yu et al. 2023). LPS, a Gram‐negative bacterial cell wall component, is used to replicate ALI patient symptoms by elevating inflammatory cell activation and promoting detrimental inflammatory mediator release (Guo, Cui, Tang, Yan, et al. 2023). It also suppresses hepatic antioxidant enzyme activities and facilitates harmful substance accumulation, accelerating ALI development (Guo, Cui, Tang, Yan, et al. 2023; Guo, Cui, Tang, Zhang, et al. 2023). Consequently, LPS is utilized to establish ALI models for assessing hepatoprotective effects of new drugs or natural active substances (Miao et al. 2023; Xu et al. 2021). Although some drugs are widely used to treat ALI, their long‐term consumption induces adverse reactions (Guo, Mao, et al. 2022). Hence, identifying safer and more effective natural bioactive substances to improve ALI symptoms is imperative.

Previous research has provided preliminary evidence that broccoli seed extract (BSE) can ameliorate liver injury (Mao et al. 2023). Furthermore, *Lacticaseibacillus paracasei* CCFM1221 was isolated and significantly enhanced the production of sulforaphane in vitro (Table S1). Based on these findings, it was hypothesized that *L. paracasei* CCFM1221 could augment the hepatoprotective effects of BSE by promoting the in vivo bioconversion of glucoraphanin to sulforaphane. To test this hypothesis, an LPS‐induced ALI mouse model was established and systematically investigated the effects of BSE supplementation, both with and without co‐administration of *L. paracasei* CCFM1221. This analysis focused on key parameters including inflammatory responses, oxidative stress, the expression of genes associated with ALI, and the composition of the gut microbial community. This approach aims to elucidate the pivotal role of this probiotic strain in enhancing the hepatoprotective efficacy of a natural product and to uncover the potential microbial mechanisms involved.

## 2 Materials and Methods

### 2.1 Reagents and Materials

Antioxidant enzyme kits were provided by Jiancheng Biotechnology Co. Ltd. (Nanjing, Jiangsu Province, China). Inflammatory cytokine kits were supplied by R&D Systems Co. Ltd. (MN, USA). BSE containing 20% glucoraphanin was sourced from Pioneer Herb Industrial Co. Ltd. (Ganzhou, Jiangxi Province, China). Glucoraphanin and sulforaphane were obtained from MedChemExpress (Shanghai, China). *L. paracasei* CCFM1221 was procured from the Culture Collection of Food Microorganisms (CCFM) in Jiangnan University (Wuxi, Jiangsu Province, China).

### 2.2 Animal Studies

Forty male C57Bl/6 mice (7 weeks old, 19 ± 1 g) were acquired from SLAC Laboratory Animal Co. Ltd. (Hangzhou, China), housed in the Experimental Animal Center of Jiangnan University with approval from the Ethics Committee (No. 20201115c0701230 [309]). After a 7‐day adaptation period, mice were divided into five groups: control, model, BSE, CCFM1221, and BSE + CCFM1221. The detailed experimental protocols for each group were summarized in Table 1. At experiment's end, control group mice were intraperitoneally injected with 0.2 mL normal saline after a 4‐h fast; other groups received 0.2 mL LPS (5 mg/kg). This dosage of LPS was selected based on a previous study by Guo et al. to ensure consistent ALI model establishment (Guo, Cui, Tang, Yan, et al. 2023; Guo, Cui, Tang, Zhang, et al. 2023). Six hours post‐LPS treatment, blood was collected from all mice via retro‐orbital puncture; livers were harvested and weighed; colonic contents were collected.

**TABLE 1:** Grouping information of animal.

Group	Intervention measures	At experiment's end, intraperitoneal injection treatment for 6 h (after a 4‐h fast)
Control	0.2 mL of normal saline	0.2 mL normal saline
Model	0.2 mL of normal saline	0.2 mL LPS (5 mg/kg)
BSE	370 mg/kg/day BSE	0.2 mL LPS (5 mg/kg)
CCFM1221	10^9^ CFU/day *L. paracasei* CCFM1221	0.2 mL LPS (5 mg/kg)
BSE + CCFM1221	370 mg/kg/day BSE and 10^9^ CFU/day *L. paracasei* CCFM1221	0.2 mL LPS (5 mg/kg)

### 2.3 Glucoraphanin and Sulforaphane Analyses

The colonic contents of glucoraphanin and sulforaphane were measured with minor modifications to previously reported methods (Wu, Cui, et al. 2023; Wu, Zuo, et al. 2023). Briefly, samples were freeze‐dried and weighed. Then 0.1 g of sample was mixed with 0.9 mL extraction liquid (acetonitrile; methanol: water = 2:2:1), followed by centrifugation (12,000 × *g* for 10 min at 25°C) to collect the supernatant. Next, 0.4 mL supernatant was transferred to a new tube and freeze‐dried using a refrigerated centrifuge. The residues were reconstituted in 0.2 mL of 80% acetonitrile for analysis using UHPLC Q Exactive MS.

### 2.4 Biochemical Analyses

Serum was collected from each mouse via centrifugation (3000 × *g* for 10 min at 25°C), and levels of (alanine transaminase) ALT, AST, and (alkaline phosphatase) ALP were measured using a blood analyzer (Toshiba, Tokyo, Japan). Liver tissue homogenates were prepared with normal saline; supernatants were collected for measuring concentrations of TNF‐α, IL‐1β, IL‐6, IL‐10 using commercial kits (R&D Systems Co. Ltd., MN, USA), as well as MDA levels and activities of SOD, CAT, and GSH‐Px using kits from Jiancheng Biotechnology Co. Ltd. (Nanjing, China).

### 2.5 Histological Analysis

Liver samples were fixed in 4% paraformaldehyde for 12 h before being embedded in paraffin. Paraffin sections were cut at 3 μm thickness and stained with hematoxylin and eosin (H&E) as well as Alcian blue‐periodic acid Schiff (AB‐PAS). Colon tissue images were captured using a 3DHISTECH Pannoramic SCAN (Budapest, Hungary).

### 2.6 Real‐Time Quantitative PCR Analysis

Hepatic RNA from each animal was extracted using Trizol reagent (Sangon Biotech, Shanghai, China) and subsequently reverse‐transcribed into cDNA with a commercial kit (Vazyme, Nanjing, China), adhering to the manufacturer's protocol. The mRNA expression levels of target genes were quantified by Real‐Time PCR (Bio‐Rad, Hercules, CA, USA) (Table S2). The expression of target genes was calculated and normalized using the 2^−ΔΔCT^ method.

### 2.7 Immunofluorescence Analysis

Immunofluorescence analysis of liver tissue was conducted in accordance with previously published methods (Li et al. 2023). Briefly, samples were incubated with primary antibodies at 4°C for 12 h, washed with phosphate‐buffered saline (PBS), and then blocked with 5% BSA for 30 min. Subsequently, the samples were incubated with secondary antibodies at 4°C for 12 h and washed with PBS. The sections were examined and imaged using a 3DHISTECH Pannoramic MIDI digital slide scanner (Budapest, Hungary).

### 2.8 Gut Microbiota Analysis

Total bacterial DNA was extracted using a DNA isolation kit (MP Biomedicals, Shanghai, China) as per the manufacturer's instructions. Amplification of the 16S rRNA gene (V3‐V4 region) was performed using 338F/806R primers. The amplified product was verified by 2% agarose gel electrophoresis, and the target fragment was purified using an AxyPrep DNA Gel Extraction Kit (Hangzhou, China). Sequencing of the purified amplicons was carried out on an Illumina Miseq PE300 platform at Jiangnan University. The raw data were processed and filtered by QIIME (v 2.0), then classified into operational taxonomic units (OTUs) based on a similarity threshold of over 97%. Alpha diversity indices (including Chao1, Shannon, Ace, and Simpson indices) of the gut microbiota were calculated, and principal component analysis (PCA) and partial least squares discriminant analysis (PLS‐DA) were performed using the online platform MetaboAnalyst (https://www.metaboanalyst.ca/).

### 2.9 Statistical Analysis

All data are presented as mean ± SD and were analyzed using GraphPad Prism 7.0. Statistical significance was determined by one‐way analysis of variance (ANOVA) followed by Duncan's multiple range test. Significant differences compared to the Model group are indicated by asterisks, where *p < 0.05, **p < 0.01, ***p < 0.001, and ****p < 0.0001. The notation “ns” indicates no significant difference.

## 3 Results

### 3.1 The Effect of BSE and *L. paracasei* CCFM1221 on Body Weight, Energy Intake, and Liver Index

**FIGURE 1:**
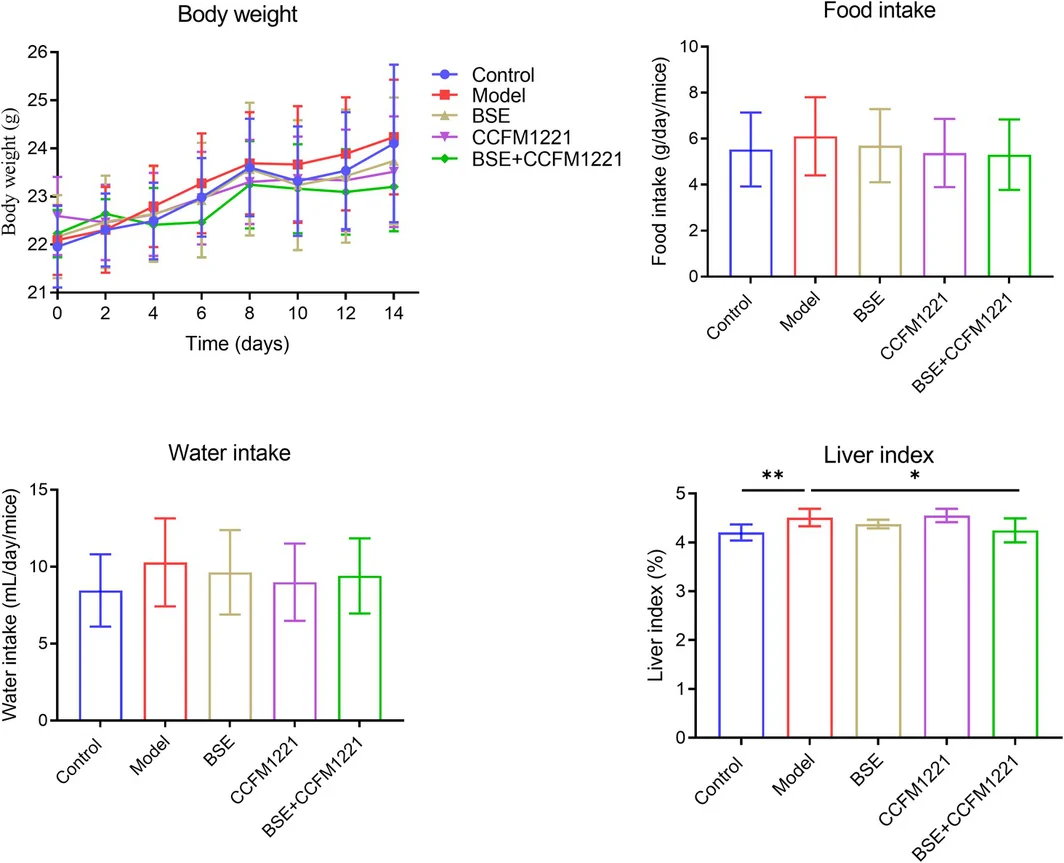
Influences of BSE and *L. paracasei* CCFM1221 on body weight, food intake, water intake, and liver index. The ‘Model’ refers to the ALI model induced by LPS.

Throughout the experiment, the body weight of each mouse was measured and recorded daily, and the food and water intake for each cage was monitored, as depicted in Figure 1. The body weight of mice in all five groups increased gradually, with no significant difference in final body weight observed among the groups (*p* > 0.05). Similarly, there were no significant differences in food and water consumption among the groups (*p* > 0.05). Compared to the control group, LPS treatment significantly increased the liver index (*p* < 0.05). Oral administration of BSE and *L. paracasei* CCFM1221 slightly reduced the liver index in LPS‐treated mice, although not significantly (*p* > 0.05); however, the liver index in the BSE group was notably lower than that in the CCFM1221 group. Intriguingly, the combination of BSE and *L. paracasei* CCFM1221 significantly counteracted the LPS‐induced increase in liver index (*p* < 0.05).

### 3.2 *L. paracasei* CCFM1221 Promotes the Hepatoprotective Effect of BSE on ALI Mice

**FIGURE 2:**
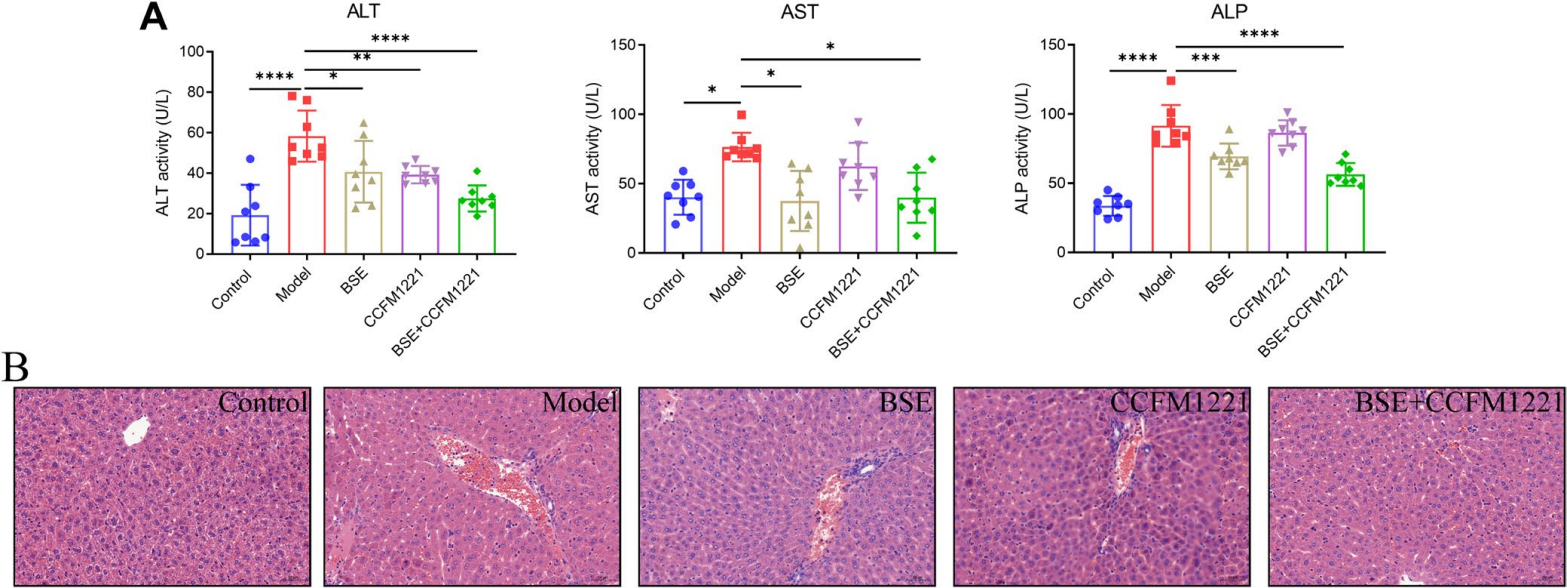
Modulation of liver injury in ALI mice by BSE and *L. paracasei* CCFM1221. (A) Activity of serum ALT, AST, and ALP; (B) H&E staining of liver tissues. The ‘Model’ refers to the ALI model induced by LPS.

The serum ALT, AST, and ALP activities were detected to assess the hepatoprotective effect of BSE and *L. paracasei* CCFM1221 in ALI mice. As illustrated in Figure 2A, LPS treatment significantly elevated serum ALT, AST, and ALP levels compared with the control group (*p* < 0.05), indicating successful establishment of the ALI model. Compared with the model group, *L. paracasei* CCFM1221 significantly reduced serum AST activity (*p* < 0.05), but did not significantly affect serum ALT and ALP activities (*p* > 0.05). However, serum ALT, AST, and ALP activities in the BSE and BSE + CCFM1221 groups were significantly lower than those in the model group (*p* < 0.05), particularly in the BSE + CCFM1221 group.

H&E staining facilitated direct observation of liver histopathological changes to evaluate the protective effect of probiotics on LPS‐induced ALI. The H&E results revealed central vein enlargement, hepatocyte swelling, loose cytoplasm, apoptosis, mild inflammatory infiltration, and hemorrhage in the model group (Figure 2B). Pretreatment with BSE, *L. paracasei* CCFM1221, or their combination ameliorated these liver histopathological alterations. Notably, the BSE + *L. paracasei* CCFM1221 group showed marked recovery in hepatocytes and hepatic cord lesions, with reduced cellular necrosis and inflammatory cell infiltration.

### 3.3 *L. paracasei* CCFM1221 Enhances the Anti‐Inflammatory Effect of BSE in ALI Mice

**FIGURE 3:**
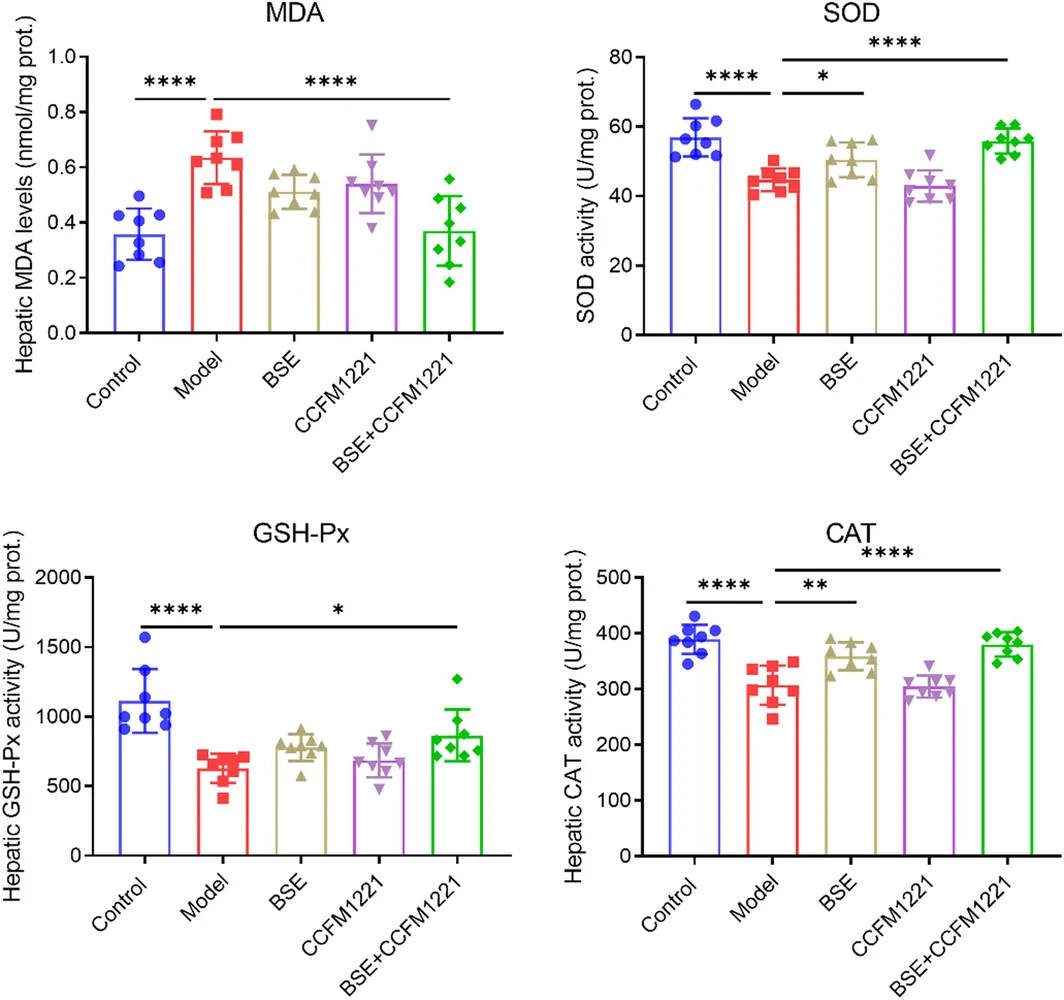
Effects of BSE and *L. paracasei* CCFM1221 on hepatic inflammatory cytokines in ALI mice. The ‘model’ refers to the ALI model induced by LPS.

Inflammation is a prominent feature of ALI; thus, hepatic inflammatory cytokines were measured in mice from all five groups (Figure 3). The hepatic levels of TNF‐*α*, IL‐1*β*, and IL‐6 in the model group were significantly elevated compared with the control group (*p* < 0.05), reaching 55.40 ± 11.48, 13.17 ± 2.38, and 11.75 ± 1.04 pg/mg protein, respectively. Additionally, LPS treatment significantly reduced hepatic IL‐10 levels compared with the control group (*p* < 0.05), down to 30.79 ± 6.39 pg/mg protein. Oral administration of *L. paracasei* CCFM1221 alone did not significantly suppress LPS‐induced changes in hepatic inflammatory cytokines (*p* > 0.05). However, BSE intervention effectively suppressed LPS‐induced elevations in hepatic TNF‐*α* and IL‐6 levels (*p* < 0.05), but not IL‐1*β* and IL‐10 levels (*p* > 0.05). Remarkably, combined treatment with BSE and *L. paracasei* CCFM1221 significantly decreased hepatic TNF‐*α*, IL‐1*β*, and IL‐6 levels while significantly increasing IL‐10 levels in LPS‐treated mice (*p* < 0.05). These findings suggest that the anti‐inflammatory effect of combined BSE and *L. paracasei* CCFM1221 intervention surpasses that of either intervention alone.

### 3.4 *L. paracasei* CCFM1221 Augments the Antioxidant Effect of BSE in ALI Mice

**FIGURE 4:**
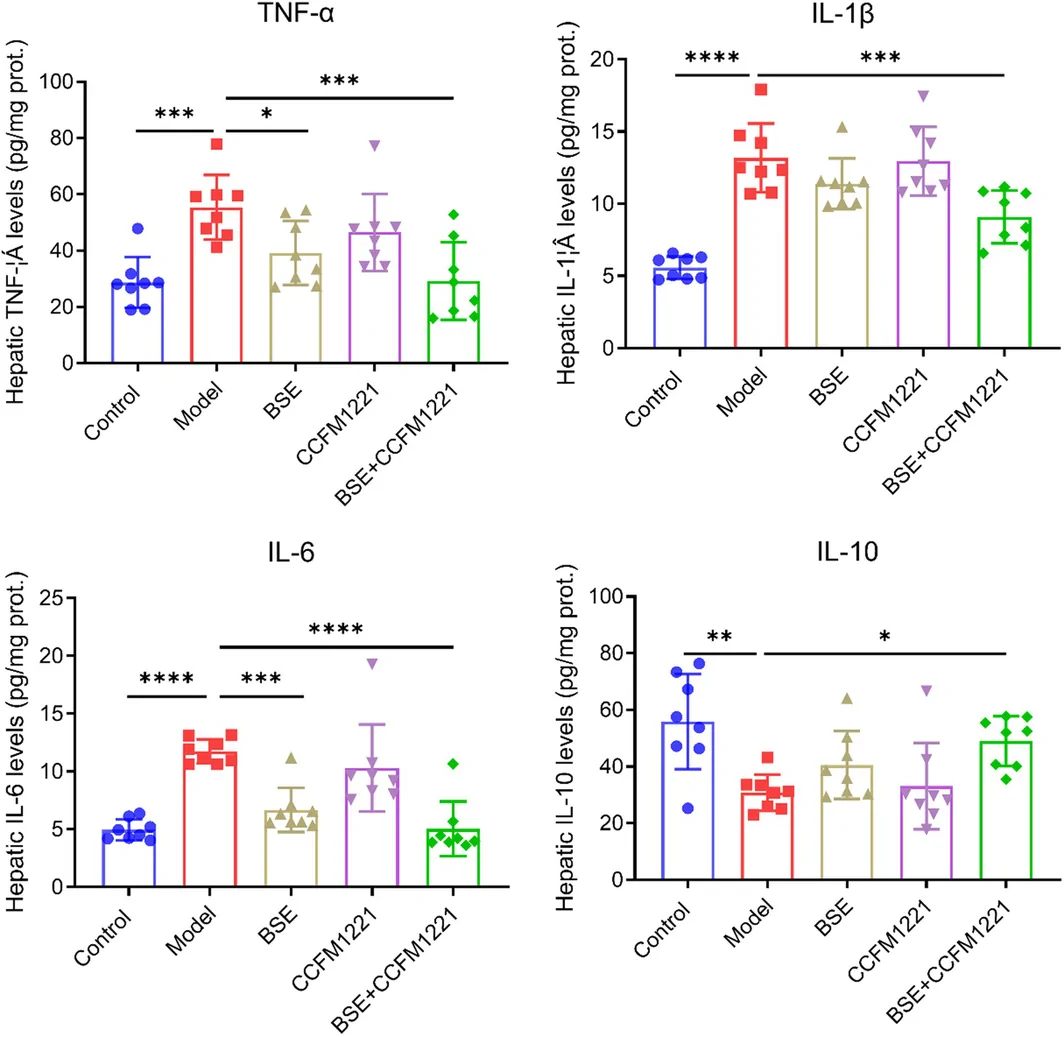
Effects of BSE and *L. paracasei* CCFM1221 on hepatic oxidative stress in ALI mice. The ‘Model’ refers to the ALI model induced by LPS.

Oxidative stress, resulting from an accumulation of reactive oxygen species, plays a crucial role in ALI pathogenesis and progression. To evaluate the hepatoprotective effect of BSE and *L. paracasei* CCFM1221 interventions—both combined and individual—hepatic levels of MDA, SOD, GSH‐Px, and CAT were measured in each group of mice. As shown in Figure 4, hepatic MDA levels were significantly increased in the model group compared with the control group (*p* < 0.05). In contrast to the model group, hepatic MDA levels were significantly reduced in both the BSE and BSE + CCFM1221 groups (*p* < 0.05), but not in the CCFM1221 group alone (*p* > 0.05). Specifically, hepatic MDA levels in the BSE + CCFM1221 group decreased from 0.64 ± 0.10 to 0.37 ± 0.13 nmol/mg protein. Furthermore, LPS treatment significantly decreased hepatic SOD, GSH‐Px, and CAT activities compared with the control group (*p* < 0.05), indicating that LPS disrupts oxidative stress balance. Oral administration of BSE significantly increased hepatic CAT activity in LPS‐treated mice (*p* < 0.05), but did not significantly affect hepatic SOD and GSH‐Px activities (*p* > 0.05). No significant differences were observed between the model and CCFM1221 groups for hepatic SOD, GSH‐Px, and CAT activities (*p* > 0.05). Notably, combined intervention with BSE and *L. paracasei* CCFM1221 significantly prevented LPS‐induced reductions in hepatic SOD, GSH‐Px, and CAT activities (*p* < 0.05). These results indicate that combined treatment with BSE and *L. paracasei* CCFM1221 effectively mitigates abnormal oxidative stress in LPS‐induced ALI mice.

### 3.5 *L. paracasei* CCFM1221 Facilitates the Regulatory Effect of BSE on mRNA Expression of Genes

**FIGURE 5:**
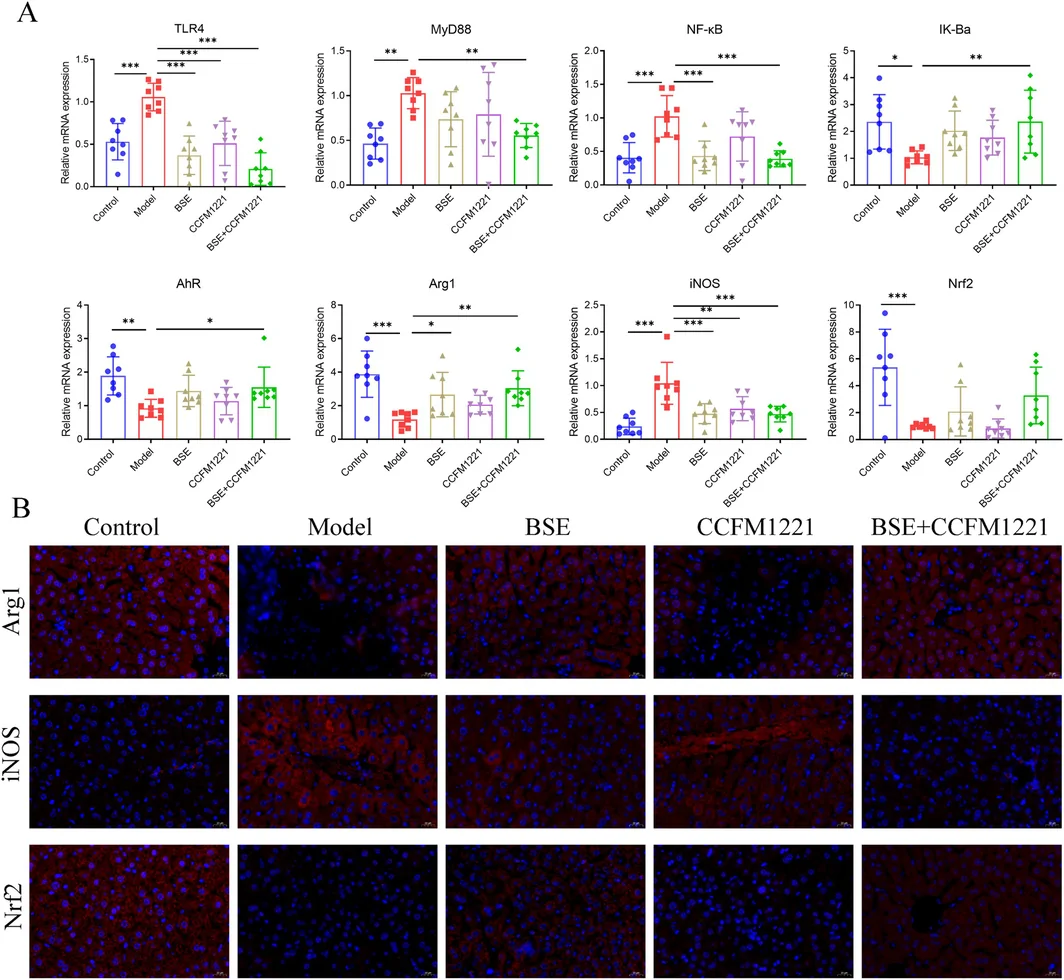
Influences of BSE and *L. paracasei* CCFM1221 on gene expression in ALI mice. (A) mRNA expression of genes; (B) Immunofluorescent analysis. The ‘Model’ refers to the ALI model induced by LPS.

Compared with the control group, the model group exhibited significant upregulation in the mRNA expression of hepatic TLR4, MyD88, NF‐κB, and iNOS (*p* < 0.05), while the expression of hepatic IK‐B*α*, AhR, Arg1, and Nrf2 was significantly downregulated (*p* < 0.05) (Figure 5A). Oral administration of BSE significantly downregulated the mRNA expression of hepatic TLR4, MyD88, NF‐κB, and iNOS and upregulated the expression of hepatic IK‐B*α* and Arg1 in ALI mice (*p* < 0.05), but it did not significantly affect the mRNA expression of hepatic AhR and Nrf2 (*p* > 0.05). Additionally, oral administration of *L. paracasei* CCFM1221 significantly altered the mRNA expression of hepatic TLR4, IK‐B*α*, and iNOS in ALI mice (*p* < 0.05). Notably, the combination of BSE and *L. paracasei* CCFM1221 significantly counteracted LPS‐induced changes in the mRNA expression of hepatic TLR4, MyD88, NF‐κB, iNOS, IK‐B*α*, AhR, Arg1, and Nrf2 (*p* < 0.05). Furthermore, immunofluorescence analysis corroborated that LPS treatment markedly decreased the expression of hepatic Arg1 and Nrf2 and increased the expression of hepatic iNOS (Figure 5B). However, both combined and individual treatments with BSE and *L. paracasei* CCFM1221 mitigated these changes to a certain extent.

### 3.6 *L. paracasei* CCFM1221 Promotes the Conversion of Glucoraphanin in ALI Mice

**FIGURE 6:**
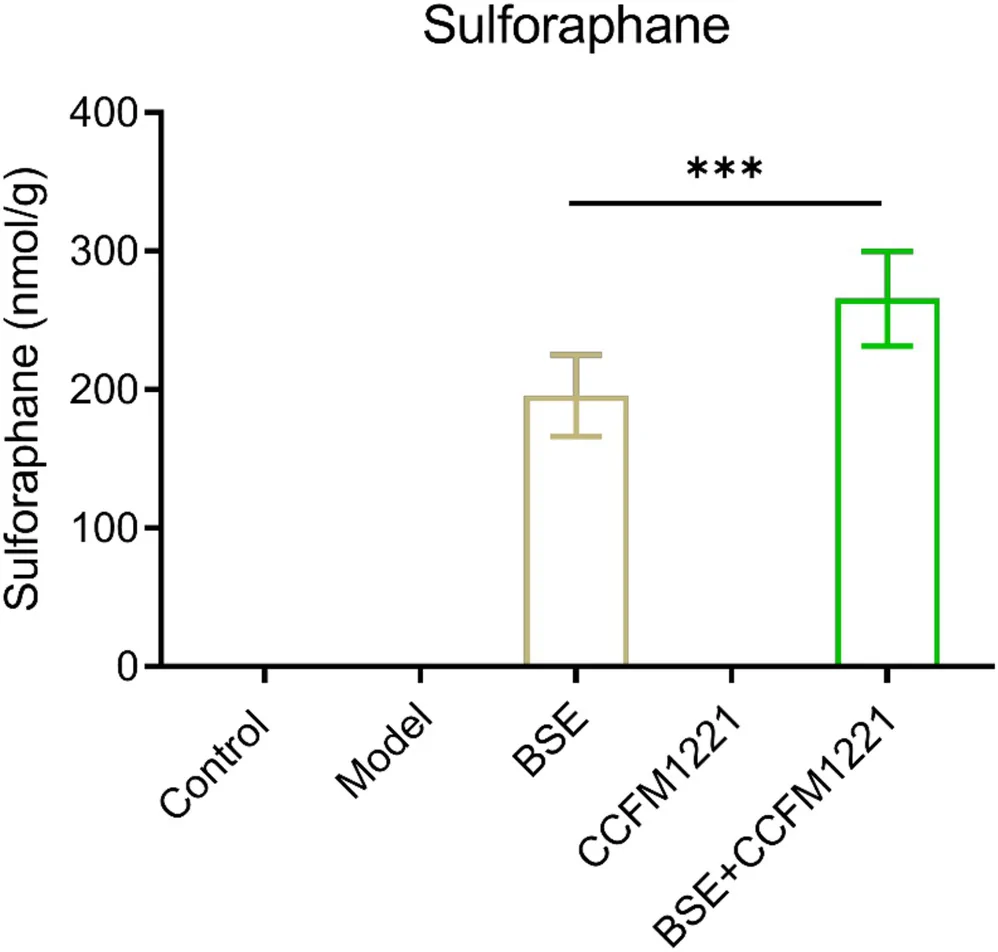
Supplementation with *L. paracasei* CCFM1221 increased colonic sulforaphane content in ALI mice. The ‘model’ refers to the ALI model induced by LPS. Administration of BSE and BSE combined with *L. paracasei* CCFM1221 resulted in colonic sulforaphane concentrations of 195.63 ± 29.60 and 265.70 ± 34.29 nmol/g.

Sulforaphane is the principal metabolite in the conversion of glucoraphanin and plays a critical role in maintaining liver function. Therefore, the content of colonic sulforaphane in the five groups was measured (Figure 6). Sulforaphane was undetectable in the intestines of mice from the control, model, and CCFM1221 groups. However, oral administration of BSE increased colonic sulforaphane content to 195.63 ± 29.60 nmol/g. Compared with the BSE group, supplementation with *L. paracasei* CCFM1221 significantly increased colonic sulforaphane content in ALI mice with BSE intervention (*p* < 0.05), reaching 265.70 ± 34.29 nmol/g. These results suggest that *L. paracasei* CCFM1221 effectively enhances the conversion of colonic glucoraphanin.

### 3.7 The Effect of BSE and *L. paracasei* CCFM1221 on Gut Microbiota in ALI Mice

**FIGURE 7:**
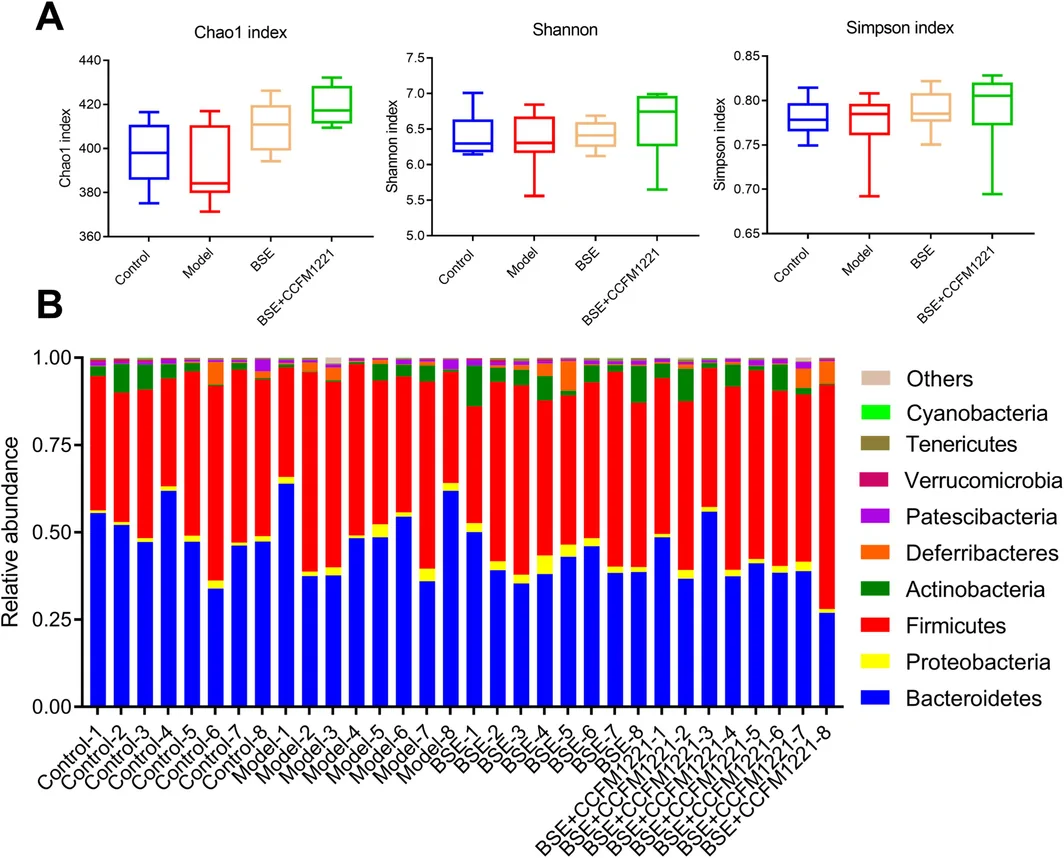
BSE and *L. paracasei* CCFM1221 modulates gut microbiota in ALI mice. (A) Chao1, Shannon index, and Simpson indices; (B) Composition of gut microbiota at phylum level. The ‘model’ refers to the ALI model induced by LPS.

The impact of BSE and *L. paracasei* CCFM1221 on gut microbiota in ALI mice was assessed using 16S rDNA sequencing. As depicted in Figure 7A, no significant differences were observed in Chao1, Shannon, and Simpson indices among the four groups (*p* > 0.05). At the phylum level, oral administration of BSE slightly increased the relative abundance of *Proteobacteria*, *Actinobacteria*, *Deferribacteres*, *Verrucomicrobia*, *Tenericutes*, *Cyanobacteria*, and slightly decreased the relative abundance of *Bacteroidetes* compared with the model group (Figure 7B and Figure S1). Furthermore, the combination of BSE and *L. paracasei* CCFM1221 slightly reduced the relative abundance of *Bacteroidetes*, *Proteobacteria*, *Verrucomicrobia*, and *Cyanobacteria* and slightly increased the relative abundance of *Actinobacteria* and *Tenericutes* compared with the model group.

**FIGURE 8:**
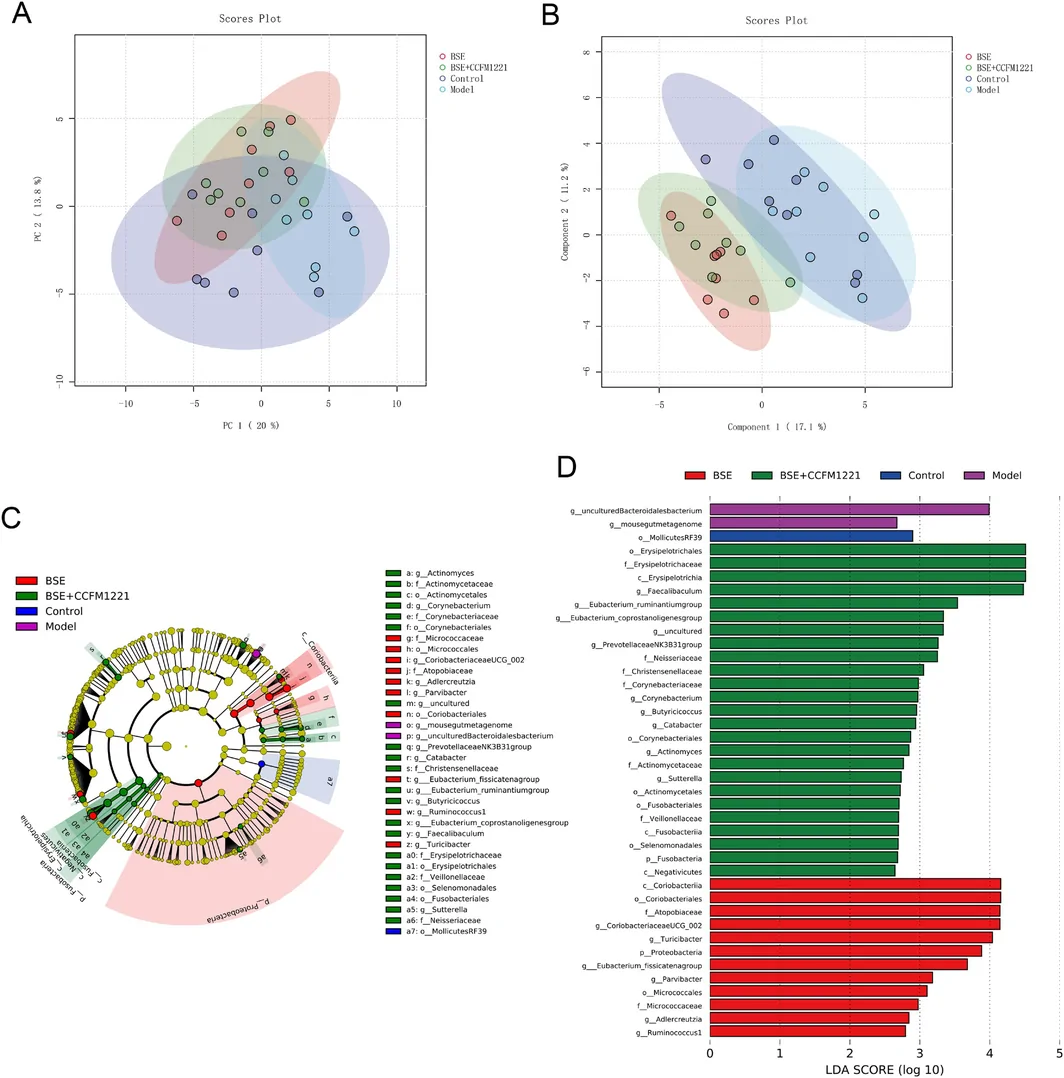
Influences of BSE and *L. paracasei* CCFM1221 on gut microbiota in ALI mice at genus levels. (A) PCA plot showing the overall structural differences in the gut microbial community among groups. A greater separation indicates a more distinct microbial profile; (B) PLS‐DA analysis, a supervised model, further validating the distinct clustering of microbial communities in response to different treatments; (C) Cladogram representing the taxonomic hierarchical structure of the gut microbiota. Nodes in different colors represent microbial lineages that were significantly enriched in the corresponding treatment group; (D) Discriminant analysis effect size (LEfSe) analysis identifying specific bacterial taxa. The ‘model’ refers to the ALI model induced by LPS. “p_” indicates Phylum, “c_” indicates Class, “o_” indicates Order, “f_” indicates Family, and “g_” indicates Genus.

PCA and PLS‐DA plots were utilized to elucidate the effect of BSE and *L. paracasei* CCFM1221 on gut microbiota (Figure 8A,B). The PCA and PLS‐DA analyses indicated that gut microbiota composition in the model group was similar to that in the control group, suggesting that intraperitoneal injection of LPS did not alter gut microbiota composition within 6 h before sacrifice. However, distinct clustering was observed for the BSE, BSE + CCFM1221, and model groups, indicating substantial shifts in gut microbiota composition for mice in the BSE and BSE + CCFM1221 groups. To further elucidate the impact of BSE and *L. paracasei* CCFM1221 intervention on gut microbiota composition in ALI mice, linear discriminant analysis effect size (LEfSe) was employed to identify the predominant genera in each group (Figure 8C,D). The control group was characterized by an uncultured Bacteroidales bacterium and mouse gut metagenome at the genus level. The BSE group was enriched with genera such as *Faecalibaculum*, *Eubacterium_ruminantium* group, *Eubacterium_coprostanoligenes* group, *Prevotellaceae* NK3B31 group, *Corynebacterium*, *Butyricicoccus*, *Catabacter*, *Actinomyces*, and *Sutterella*. Conversely, the BSE + CCFM1221 group was characterized by Coriobacteriaceae_UCG‐002, *Turicibacter*, *Eubacterium_fissicatena* group, *Parvibacter*, *Adlercreutzia*, and Ruminococcus_1 at the genus level. These findings indicate that both combined and individual supplementation with BSE and *L. paracasei* CCFM1221 altered gut microbiota composition in ALI mice.

## 4 Discussion

This study advances the current understanding of probiotic‐enhanced hepatoprotection by demonstrating a novel synergistic strategy: combining BSE with a specific sulforaphane‐producing probiotic strain, *L. paracasei* CCFM1221. Unlike previous studies that focused on BSE alone, this study provides direct quantitative evidence that this co‐administration significantly increases colonic sulforaphane levels by approximately 36%, establishes a causal link between the probiotic and enhanced bioactive compound generation, and reveals a dual functional remodeling of the gut microbiota by simultaneously enriching both short‐chain fatty acid‐producing bacteria and sulforaphane‐converting microbiota. Furthermore, the underlying molecular mechanisms were elucidated, demonstrating that the combined intervention exerts hepatoprotective effects through dual modulation of the TLR4/NF‐κB inflammatory pathway and the Nrf2 antioxidant pathway, thereby bridging the gap between probiotic‐mediated microbial metabolism and host physiological responses.

The liver is a crucial organ responsible for regulating energy metabolism and controlling drug/toxicant metabolism; its high susceptibility to injury makes it prone to fibrosis, cirrhosis, and other diseases (Roma et al. 2023). ALI can lead to liver failure and pose a significant threat to human life; its clinical management is challenging due to its complex pathological mechanisms and absence of preexisting liver diseases. Therefore, intraperitoneal injection with LPS is utilized to establish an ALI model to explore the effect of *L. paracasei* CCFM1221 on the hepatoprotective effect of BSE. These findings demonstrate that *L. paracasei* CCFM1221 enhances the anti‐inflammatory and antioxidant effects of BSE by modulating TLR4/NF‐κB and Nrf2 pathways in LPS‐induced mice, thereby preventing ALI onset/progression.

Serum levels of ALT, AST, and ALP are widely recognized clinical biomarkers for assessing liver injury severity. Previous research has shown that serum ALT, AST, and ALP levels significantly increase following intraperitoneal injection of LPS into mice after 6 h (Havira et al. 2020), corroborating our results. ALT is predominantly found in hepatocyte cytoplasm; its elevation indicates cell membrane damage (Guo et al. 2018). AST is mainly located in hepatocyte mitochondria; its elevation signifies mitochondrial damage (Guo, Cao, et al. 2022; Guo, Mao, et al. 2022). Moreover, serum ALP levels are indicative of liver function as they increase when hepatocyte membranes are compromised (Shu et al. 2023). In this study, both combined and individual treatments with BSE and *L. paracasei* CCFM1221 effectively reduced serum ALT, AST, and ALP levels—particularly notable with combined treatments—suggesting they prevent LPS‐induced liver function deterioration. Additionally, intraperitoneal injection with LPS significantly increased liver index in mice (Hua et al. 2023).

LPS‐induced ALI is a widely used animal model as it activates macrophages to produce massive inflammatory cytokines (Dong et al. 2022). Studies have confirmed that LPS activates NF‐κB by binding to TLR4 receptors and modulating MyD88 expression (Fu et al. 2021; Xue et al. 2021). However, NF‐κB activation and nuclear translocation are regulated by hepatic IK‐Bα levels to some extent. Elevated IK‐Bα expression is known to inhibit inflammatory cytokine production via NF‐κB pathways, including TNF‐*α*, IL‐1*β*, and IL‐6 (Guo, Cui, Tang, Yan, et al. 2023; Guo, Cui, Tang, Zhang, et al. 2023). TNF‐α is a critical cytokine mediator whose overproduction enhances pro‐inflammatory and proapoptotic effects across various cell types (Zhang, Wei, et al. 2023; Zhang, Cui, et al. 2023). TNF‐*α* can also promote IL‐6 and IL‐1*β* production. Excessive IL‐6 exacerbates local inflammation and impairs organ functions by disrupting energy metabolism (Tang et al. 2005). Previous studies have suggested that sulforaphane suppresses inflammatory cytokine production by modulating NF‐κB pathways (Liu et al. 2017). This study indicates that combined treatment with BSE and *L. paracasei* CCFM1221 effectively inhibits mRNA expression of hepatic TLR4, MyD88, NF‐κB, iNOS in ALI mice while promoting mRNA expression of hepatic IK‐B*α* and Arg1—potentially due to *L. paracasei* CCFM1221's role in enhancing glucoraphanin's biotransformation to sulforaphane. iNOS and Arg1 are expressed predominantly in pro‐inflammatory M1 macrophages and anti‐inflammatory M2 macrophages respectively (Guo, Cao, et al. 2022; Guo, Mao, et al. 2022). These findings confirm that combined treatment with BSE and *L. paracasei* CCFM1221 offers greater hepatoprotective effects than individual treatments alone—likely associated with enhanced biotransformation of glucoraphanin to sulforaphane.

Oxidative stress, caused by the disruption of the balance between the production and degradation of reactive oxygen species, is a major response to LPS‐induced liver injury. The elimination of reactive oxygen species is strongly related to the activity of antioxidant enzymes, such as SOD, GSH‐Px, and CAT (Wang et al. 2023). Activated SOD and CAT can reduce the accumulation of reactive oxygen species, a major factor stimulating overloaded oxidative stress (Li, Guo, et al. 2019; Li, Mei, et al. 2019). Additionally, SOD elevates H_2_O_2_ content, which GSH‐Px then degrades into non‐toxic substances (oxygen and water) (Lv et al. 2022). Furthermore, lipid peroxidation is regarded as a significant cause of LPS‐induced liver injury, with MDA acting as the final product of lipid peroxidation, widely used to diagnose the degree of oxidative damage (Janero 1990). In this study, both combined and individual treatments with BSE and *L. paracasei* CCFM1221 increased antioxidant enzyme (SOD, GSH‐Px, and CAT) activity and reduced MDA levels, especially in the BSE + CCFM1221 group. Additionally, Nrf2 is regarded as a stress‐responsive monitor that controls the expression of cytoprotective proteins to prevent cell injury induced by oxidative stress and inflammation (Mao et al. 2023). Sulforaphane is confirmed to promote the dissociation of Nrf2 from Keap1 and the translocation of Nrf2 to the nucleus, which prevents the expression of pro‐inflammatory cytokine genes (Wu et al. 2017). *L. paracasei* CCFM1221 was found to promote the production of colonic sulforaphane in ALI mice with BSE supplementation, which may be useful for activating the Nrf2 pathway.

Growing evidence indicates that probiotics could prevent the development of ALI by shifting gut microbiota composition (Yan et al. 2023). Therefore, gut microbiota is considered a potential target for alleviating ALI development. In this study, *Faecalibaculum*, *Eubacterium_ruminantium* group, *Eubacterium_coprostanoligenes* group, *Prevotellaceae* NK3B31 group, *Corynebacterium*, and *Butyricicoccus* were enriched in the BSE group. *Faecalibaculum*, a gram‐positive obligate anaerobe, is reported to reduce gut inflammatory monocytes (Zagato et al. 2020). As short‐chain fatty acids (SCFA)‐producing bacteria, *Eubacterium_ruminantium* group, *Eubacterium_coprostanoligenes* group, *Corynebacterium*, and *Butyricicoccus* have been reported to be positively correlated with the regulation of inflammatory responses and oxidative stress (Wu, Shi, et al. 2020; Yi et al. 2021; Zhao et al. 2023). The increases in SCFA‐producing bacteria might be associated with BSE‐containing polysaccharides. However, Coriobacteriaceae_UCG‐002, *Turicibacter*, *Eubacterium_fissicatena* group, *Parvibacter*, *Adlercreutzia*, and *Ruminococcus*_1 were enriched in the BSE + CCFM1221 group. Coriobacteriaceae_UCG‐002, belonging to the Actinobacteria phylum, is strongly related to glucose metabolism regulation (Liu et al. 2018). *Turicibacter* and *Parvibacter*, SCFA‐producing bacteria, have been confirmed to possess anti‐inflammatory and immunomodulatory effects (Wu, Yang, et al. 2020; Wu, Zuo, et al. 2023). *Ruminococcus*, a strict anaerobe in healthy individuals' guts, is reported to prevent obesity development by regulating energy metabolism (Del Chierico et al. 2017). Increases in *Eubacterium_fissicatena* group abundance helped elevate colonic sulforaphane levels (Wu, Cui, et al. 2023). A previous study also suggested that gut bacteria have the capacity to metabolize glucosinolates into sulforaphane (Liou et al. 2020), which was evident in the colonic sulforaphane content of the BSE/BSE + CCFM1221 group in this study. Therefore, it is speculated that *L. paracasei* CCFM1221 promotes the hepatoprotective effect of BSE on lipopolysaccharide (LPS)‐treated mice by elevating the biotransformation of glucoraphanin to sulforaphane.

Although the hepatoprotective effect of *L. paracasei* CCFM1221 in synergizing with BSE has been studied, there are still some limitations. First, although colonic sulforaphane levels were quantified, glucoraphanin concentrations in animals were not measured. Future studies incorporating comprehensive mass balance analyses of both glucoraphanin and sulforaphane in feces, colonic contents, and tissues are warranted to fully elucidate the in vivo conversion kinetics and bioavailability. Second, only single doses of BSE and *L. paracasei* CCFM1221 were evaluated, and safety assessments—including probiotic colonization dynamics and potential host–microbe interactions—were not performed. Third, these findings are derived from an acute LPS‐induced liver injury model, which may not fully recapitulate the pathophysiology of chronic liver diseases, and translational gaps remain regarding human‐equivalent dosing and clinical feasibility. Future research employing dose–response designs, chronic disease models, metagenomic sequencing, and comprehensive mass balance analyses is warranted to validate and extend these findings.

In conclusion, this study demonstrated that *L. paracasei* CCFM1221 enhances the hepatoprotective effect of BSE on LPS‐treated mice by elevating the biotransformation of glucoraphanin to sulforaphane. This primarily manifested as preventing inflammatory responses and oxidative stress by regulating TLR4/NF‐κB and Nrf2 pathways. Moreover, it was found that combined intervention with BSE and *L. paracasei* CCFM1221 not only increased the abundance of SCFA‐producing bacteria but also promoted the growth of sulforaphane‐producing bacteria. Collectively, these data offer a novel approach to improving dietary phytochemicals' bioactivity.

## Author Contributions

**Shumao Cui:** resources, project administration. **Wei Chen:** funding acquisition, project administration. **Qiuxiang Zhang:** supervision, data curation. **Jianxin Zhao:** resources, project administration. **Weiling Guo:** writing – original draft, software. **Bingyong Mao:** supervision, writing – review and editing, funding acquisition. **Xin Tang:** writing – review and editing. **Yi Wei:** writing – review and editing, validation.

## Funding

This work was supported by the Qinglan Project in Jiangsu Province, Yongjiang Talent Introduction Programme (2021C‐003‐T), and the Collaborative Innovation Center of Food Safety and Quality Control in Jiangsu Province.

## Conflicts of Interest

The authors declare no conflicts of interest.

## Supporting information


**Figure S1:** Influence of BSE and *L. paracasei* CCFM1221 on the gut microbiota at the phylum level.
**Table S1:** Transformation of glucoraphanin by *L. paracasei* CCFM1221.
**Table S2:** Primer sequences.

## Data Availability

The data presented in this study are available upon reasonable request from the corresponding author.
