# Supplementary material for: Sulforaphane‐Producing Lacticaseibacillus paracasei CCFM1221 Combining Broccoli Seed Extract Improved the Hepatoprotective Effect on Lipopolysaccharide‐Treated Mice
Source: Food Sci Nutr. 2026 Aug 24;14(8):e72271. doi: 10.1002/fsn3.72271 (PMC13504240; doi:10.1002/fsn3.72271)
Supplement: Supplementary file 1 [file FSN3-14-e72271-s001.docx]

## Sulforaphane-producing *Lacticaseibacillus paracasei* CCFM1221 combining broccoli seed extract improved the hepatoprotective effect on lipopolysaccharide-treated mice

Xin Tang^1,2^, Yi Wei^1,2^, Weiling Guo^1,2^, Qiuxiang Zhang^1,2^, Jianxin Zhao^1,2^, Wei Chen^1,2,3^, Bingyong Mao^1,2,*^, Shumao Cui^1,2,4^

^1^ State Key Laboratory of Food Science and Resources, Jiangnan University, Wuxi, 214122, China

^2^ School of Food Science and Technology, Jiangnan University, Wuxi, 214122, China

^3^ National Engineering Research Center for Functional Food, Jiangnan University, Wuxi, 214122, China

^4^ International Joint-Research Laboratory for Maternal-Infant Microbiota and Health, Jiangnan University, Wuxi, 214122, China

^*^ Corresponding author: State Key Laboratory of Food Science and Resources, Jiangnan University, Lihu Avenue 1800, Wuxi, 214122, China.

E-mail address: maobingyong@jiangnan.edu.cn (B. Mao).

**Measures**

**Glucoraphanin and Sulforaphane Analyses**

Glucoraphanin and Sulforaphane were determined using the same method as described in the main text.

**Result**

**Metabolism of glucoraphanin by *L. paracasei* CCFM1221**

Sulforaphane, a secondary metabolite derived from glucoraphanin, has been demonstrated to possess hypoglycemic, hypolipidemic, and anticancer properties. Human metabolism of glucoraphanin can occur through the consumption of BSE and other cruciferous vegetables, yet there is considerable inter-individual variability. *L. paracasei* CCFM1221 is capable of metabolizing glucoraphanin, converting 7.5% of it through fermentation in a medium containing 2 g/L glucoraphanin over a 24 h period. To enhance the metabolic capacity of *L. paracasei* CCFM1221, We optimized the nitrogen source, and the findings revealed that 5 g/L of tryptone did not enhance the metabolism of glucoraphanin by *L. paracasei* CCFM1221, yielding a conversion rate of only 8%. When the tryptone concentration in the medium was decreased by 2.5 g/L and an equivalent amount of yeast extract was added, the metabolic capacity of *L. paracasei* CCFM1221 for glucoraphanin increased to 33%. Furthermore, replacing all tryptone with an equal amount of yeast extract in the medium elevated the conversion rate to 38% (Table S1).

**Supplementary data**

**Figure S1** Influence of BSE and *L. paracasei* CCFM1221 on the gut microbiota at at the phylum level.

**Table S1** Transformation of glucoraphanin by *L. paracasei* CCFM1221

| Group | Nitrogen source | Reduction of glucoraphanin (g/L) | Conversion rate(%) |
| --- | --- | --- | --- |
| Glucoraphanin-containing mediuma^a^ | none | 0.15±0.02 | 7.5 |
| Modified medium 1 | 5g/L tryptone | 0.16±0.02 | 8 |
| Modified medium 2 | 2.5g/L yeast extract+2.5g/L tryptone | 0.66±0.05 | 33 |
| Modified medium 3 | 5g/L yeast extract | 0.76±0.04 | 38 |

^a^The glucoraphanin-containing medium comprised a phosphate buffer at pH 7.0, 0.05 mM in concentration, 2.0 g/L of glucoraphanin, 1 g/L of cysteine, and 1 g/L of ascorbic acid.

**Table S2** Primer sequences.

| Name | Forward sequences (5′-3′) | Reverse sequences (5′-3′) |
| --- | --- | --- |
| TLR4 | GGCAGCAGGTGGAATTGTAT | AGGCCCCAGAGTTTTGTTCT |
| MyD88 | GTGCCGTCGGATGGTAGTG | GACAGTGATGAACCGCAGGAT |
| Iκ-βα | ACCAACCAGCCAGAAATCG | TCACAGGCAAGGTGTAGAGGG |
| NF-κB | CGCCCCTTATCGACCACC | CCTTCTCCCAAGAGTCGTCCA |
| AhR | CATCTATCTGTGTCCTCCTTCAAGC | CCGCACTTGCTCACGGA |
| Arg1 | AGACAGCAGAGGAGGTGAAGA | CGAAGCAAGCCAAGGTTAAAG |
| iNOS | CTTGGAGCGAGTTGTGGATTGTC | TAGGTGAGGGCTTGGCTGAGT |
| Nrf2 | CCTCCGCTGCCATCAGTCAGT | TCGGCTGGGACTCGTGTTCA |
| GAPDH | AGGTCGGTGTGAACGGATTTG | GGGGTCGTTGATGGCAACA |
